# Calendar

**DOI:** 10.1155/S0962935192000656

**Published:** 1992


					Calendar
Calendar
1993
29-31 January, 1993
Second International Congress on
Biological Response Modifiers (BRM)
San Diego, USA
Contact: Meetings Manager, CME Inc,
PO Box 712, Princeton Junction, NJ
08550, USA.
12-17 March, 1993
The American Academy of Allergy &
Immunology Annual Meeting
Chicago, USA
Contact: The Executive Office, AAAI,
611 East Wells Street, Milwaukee,
Wisconsin 53202, USA
Tel: + 414 272 6071
1-4 April, 1993
2nd International Symposium on the
Immunotherapy of the Rheumatic
Diseases, Brighton, UK
Contact: Professor G. S. Panayi, The
Rheumatology Unit, Division Medicine,
4th Floor Hunts House, UMDS, Guy's
Hospital, London SE1 9RT, UK
19-20 April, 1993
15th Meeting of BIRA Auto-immune
Diseases: Immunogenetic Mechanisms
and Strategies for Therapy
The Medical School, University of
Newcastle-Upon-Tyne, UK
Contact: Dr JA Goodacre and Dr A
Diamond, University of Newcastle
7-11 June, 1993
9th International Conference on AIDS
Berlin, Germany.
Contact: K-O Habermehl, Chairman,
Institute for Clinical and Experimental
Virology
Free University of Berlin,
Hindenburgdamm 27, D-1000 Berlin 30,
Germany
21-24 June, 1993
Congrhs International de Pneumologie
de Langue Francaise
Marrakech, Moroco
Contact: Secretariat, Congres
International de Pneumologie de
Langue Francaise, Societe Marocaine
des Maladies Respiratoires
BP 1828 Derb Ghallef, Casablanca,
Maroc.
24-25 June, 1993
Cells and Cytokines in Lung
Inflammation
Paris, France
Contact: B B Vargaftig, Unite de
Pharmacologie Cellulaire
Unite Associee Institut Pasteur,
INSERM No 285, 25 Rue du Dr Roux,
75015, Paris, France
4-10 July, 1993
XVIIth ILAR Congress of
Rheumatology
Barcelona, Spain
Contact: Viajes Iberia Congresos,
Diagonal; 523, lo 08029 Barcelona, Spain
12-15 September, 1993
26th Annual Meeting of the European
Academy of Allergology and
Clinical Immunology (EAACI)
Rotterdam, The Netherlands
Contact: ICOF/Congrex, PO Box 22358
3003 DJ Rotterdam, The Netherlands
10-15 October, 1993
1st International Congress of the
International Association of
Inflammation Societies (IAIS)
Vienna, Austria
Contact: Inflammation '93 Congress
Secretariat, ICOS Congress
Organization Service Schleifmuhlgosse
1, A-1040, Vienna, Austria
24-29 October, 1993
14th Congress of the International
Association of Asthmology
Jerusalem, Israel
Contact: Professor Glazer, Rh
Bograshov 89, Tel Aviv 63297, Israel
1994
26 June-1 July, 1994
27th Meeting of the European Academy
of Allergology and Clinical Immunology
(EAACI)/XVth International Congress
of Allergology and Clinical Immunology
(ICACI)
Stockholm, Sweden
Contact: Secretariat, ICACI XV-
EAACI'94, c/o Congrex, PO Box 5619,
S-114 86 Stockholm, Sweden
20-25 November, 1994
2nd International Congress for
Pathophysiology
Kyoto International Conference Hall,
Kyoto, Japan
Contact: Dr Toshiie Sakata, First
Department of Internal Medicine, Ooita
Medical University, 1-1 Idaigaoka
Hazamacho, Ooita-gun, Ooita, 879-55,
Japan
Tel: +81 975 49 4411
Fax: +81 975 49 4217
(C) 1992 Rapid Communications of Oxford Ltd Mediators of Inflammation. Vol 1992 4,29

				

